# Enhancing Target Tracking: A Novel Grid-Based Beetle Antennae Search Algorithm and Confusion-Aware Detection

**DOI:** 10.3390/biomimetics9090567

**Published:** 2024-09-19

**Authors:** Yixuan Lu, Chencong Ma, Dechao Chen

**Affiliations:** School of Computer Science and Technology, Hangzhou Dianzi University, Hangzhou 310018, China; 212050164@hdu.edu.cn (Y.L.); machencong@hdu.edu.cn (C.M.)

**Keywords:** tracking control, path planning, bionic algorithms, object detection, unmanned aerial vehicle

## Abstract

Unmanned aerial vehicle target tracking is a complex task that encounters challenges in scenarios involving limited computing resources, real-time requirements, and target confusion. This research builds on previous work and addresses challenges by proposing a grid-based beetle antennae search algorithm and designing a lightweight multi-target detection and positioning method, which integrates interference-sensing mechanisms and depth information. First, the grid-based beetle antennae search algorithm’s rapid convergence advantage is combined with a secondary search and rollback mechanism, enhancing its search efficiency and ability to escape local extreme areas. Then, the You Only Look Once (version 8) model is employed for target detection, while corner detection, feature point extraction, and dictionary matching introduce a confusion-aware mechanism. This mechanism effectively distinguishes potentially confusing targets within the field of view, enhancing the system’s robustness. Finally, the depth-based localization of the target is performed. To verify the performance of the proposed approach, a series of experiments were conducted on the grid-based beetle antennae search algorithm. Comparisons with four mainstream intelligent search algorithms are provided, with the results showing that the grid-based beetle antennae search algorithm excels in the number of iterations to convergence, path length, and convergence speed. When the algorithm faces non-local extreme-value-area environments, the speed is increased by more than 89%. In comparison with previous work, the algorithm speed is increased by more than 233%. Performance tests on the confusion-aware mechanism by using a self-made interference dataset demonstrate the model’s high discriminative ability. The results also indicate that the model meets the real-time requirements.

## 1. Introduction

Unmanned aerial vehicle (UAV) autonomous tracking technology [1,2] is an innovative technology based on advanced sensors, intelligent algorithms, and autonomous decision-making capabilities. Its primary objective is to enable UAVs to independently identify, track, and pursue targets without human intervention, achieving efficient and accurate target tracking. Autonomous aerial tracking faces challenging tasks in fields such as search and rescue, pursuit, photography, and surveillance. Given the urgent need for UAV autonomous tracking solutions, the real-time tracking system design is considered a fundamental and crucial issue in UAV research. To achieve autonomous tracking, the UAV real-time tracking system relies on advanced sensors and high-performance computing platforms, utilizing deep learning and artificial intelligence algorithms to rapidly identify, locate, and predict the dynamic behavior of targets. It then plans a safe and reliable path within a short time frame. Moreover, to handle unpredictable situations, the system requires highly responsive, accurate, and intelligent path planning algorithms [3,4,5].

Some state-of-the-art aerial tracking planners [6,7,8] addressing the above issues have shown significant robustness and impressive agility. However, several limitations remain with these approaches. Firstly, the system’s high hardware demands may constrain its deployment on UAV platforms with limited computational capabilities. Secondly, the path search algorithm is relatively outdated and lacks efficiency compared to more advanced contemporary methods. Additionally, while the system offers a lightweight design, its target detection capabilities are limited to a narrow range, reducing its adaptability across broader application scenarios. Finally, the system demonstrates shortcomings in handling complex environments; for instance, the presence of visually similar objects within the field of view can result in tracking failures, ultimately diminishing the overall system performance.

In this study, two significant technical innovations are proposed: a confusion-aware multi-class object detection mechanism and a grid-based path planning algorithm utilizing the beetle antennae search (BAS) approach. For object detection, a lightweight framework based on machine learning and deep learning techniques is introduced. This framework effectively tracks multiple object categories in real time and incorporates a confusion-aware mechanism, enabling it to differentiate between similar-category targets by employing feature point matching and target position estimation. This significantly enhances robustness and real-time performance, particularly in scenarios with multiple similar objects within the field of view. For path planning, a grid-based algorithm is developed that leverages the rapid convergence properties of bionic algorithms. A distance-feedback-based step-size update strategy is employed to improve the efficiency of navigation. Additionally, a dynamically constructed grid index enables secondary searches, effectively reducing the overall path length. A fallback mechanism is also implemented to assist the system in escaping local minima, ensuring adaptive and efficient tracking in complex environments.

**Figure 1:**
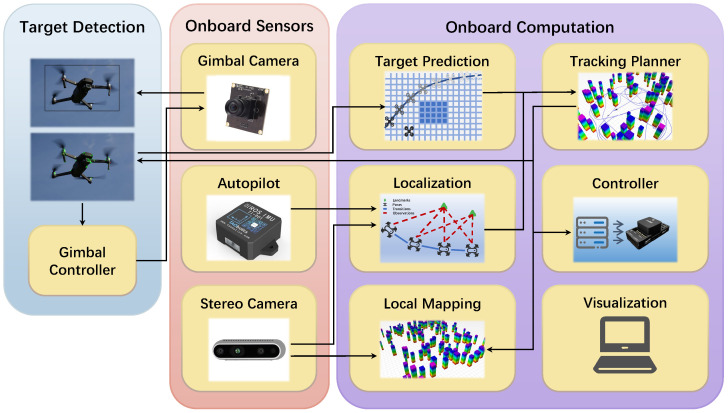
The architecture of the quadrotor system with target detection and a tracking planner.

These methods are integrated into a comprehensive UAV tracking system developed in previous work. As illustrated in Figure 1, the system consists of the following key components: (1) multi-class object detection with a confusion-aware mechanism, (2) future motion prediction for targets without explicit motion intent, (3) a grid-based BAS algorithm for the tracking path search, (4) the generation of safe and dynamically feasible tracking trajectories, and (5) an onboard UAV system equipped with omnidirectional vision capabilities. The primary focus of this work is on improving components (1) and (3), as they directly influence the system’s tracking accuracy and path planning efficiency. The contributions of this study can be summarized as follows:

- A grid-based path planning algorithm inspired by the BAS algorithm.
- A secondary search mechanism based on a dynamic grid index and a distance-based step update strategy to improve the path quality and search speed.
- A grid fallback mechanism to escape local extreme regions.
- A confusion-aware multi-class object detection and localization framework.

The remainder of this paper is organized as follows. Section 2 provides a comprehensive literature review, summarizing relevant works on UAV autonomous tracking, path planning algorithms, and object detection methods, particularly focusing on those using confusion-aware techniques and bionic algorithms. Section 3 presents the improved grid-based beetle antennae search (GBAS) algorithm and its implementation. This section also discusses the design and integration of the confusion-aware mechanism and depth information for multi-class object tracking. Section 4 details the experimental setup and evaluation results, providing insights into the performance improvements achieved by our proposed methods. Finally, Section 5 concludes the paper and suggests potential directions for future research.

## 2. Related Work

### 2.1. Neural Networks in UAV Target Tracking

In recent years, the development of neural networks has had a profound impact on UAV autonomous tracking systems [9]. The rise of deep learning technology has led to significant advancements in image recognition [10,11] and target tracking [12,13] for UAVs. Through deep learning models like convolution neural networks (CNNs) [14,15], UAVs can efficiently and accurately identify targets such as humans, vehicles, and animals from the image data captured by sensors, laying a solid foundation for subsequent tracking tasks. Additionally, deep learning methods allow UAVs to learn target motion patterns and features. By employing algorithms based on long short-term memory networks (LSTMs) [16,17] or CNNs, the tracking robustness and reliability have been improved, effectively addressing issues like rapid target changes, occlusions, and complex backgrounds. Optimized network structures and algorithms have significantly increased the speed of target recognition and tracking, meeting the real-time decision-making requirements for UAVs in dynamic environments. Through end-to-end learning, neural networks enable UAVs to directly learn from perceptual data and make autonomous decisions, reducing reliance on human intervention and achieving a higher degree of autonomy. However, the application of neural networks also brings challenges, such as increased model complexity and computational requirements. Efficient computation in embedded systems is necessary to meet the real-time demands of UAV autonomous tracking systems. Moreover, acquiring and annotating a large amount of real-world data, especially in complex and changing environments, remains a challenging aspect of training neural network models for this application.

You Only Look Once (YOLO) [18] has established itself as one of the leading algorithms for real-time object detection due to its ability to balance speed and accuracy. Since its introduction, YOLO has been extensively studied and applied in various fields, including surveillance, autonomous driving, and robotics, where real-time performance is critical. Its capability to detect multiple objects in a single frame and its efficient computation make it a preferred choice for tasks requiring high-speed detection and classification. With the growing interest in technology cross-fusion, researchers have integrated YOLO with UAV systems, forming what is known as YOLO-based UAV technology (YBUT) [19]. YBUT leverages the fast object detection capabilities of YOLO and applies them to the aerial perspectives provided by UAVs. This combination has enabled more advanced UAV applications, such as autonomous navigation, surveillance, and search-and-rescue operations, where real-time decision-making based on object detection is crucial. YBUT has demonstrated excellent performance in dynamic target detection and classification, making it particularly suitable for UAV-based moving target tracking. Many studies have shown that YBUT is capable of identifying and tracking multiple moving targets in real time, even in complex environments where targets may be occluded or where multiple objects of similar appearance are present [20,21,22]. This adaptability and real-time processing capability make YBUT a popular choice for UAV applications, particularly in dynamic and challenging environments.

### 2.2. Path Planning in UAV Target Tracking

In the field of path planning, many studies have shown that efficiency and robustness are equally important in complex environments [23,24,25]. Various bio-inspired methods [26,27] have been developed to enhance planning performance, such as the artificial bee colony (ABC) algorithm based on bee foraging behavior [28,29]; the ant colony (ACO) algorithm, which utilizes a cooperative swarm search [30,31,32]; the genetic algorithm (GA) [33,34]; and the particle swarm optimization (PSO) algorithm, suitable for large-scale problems [35,36]. These algorithms have demonstrated remarkable robustness and impressive efficiency in different scenarios. However, they still suffer from certain inefficiencies. In [37], a spherical vector-based PSO (SPSO) algorithm was proposed, which used a PSO method based on ball support to transform the path planning problem into an optimization problem. Experimental results proved the excellent performance of SPSO. The IACO-IABC was proposed in [38], which combined the ACO and ABC algorithms and had impressive stability. The goal-distance-based rapidly exploring random tree* (GDRRT*) [39] improved the traditional RRT* algorithm by introducing an intelligent sampling mechanism for the target distance and had a faster convergence speed than RRT*. The above studies are novel attempts at path planning problems using heuristic algorithms.

Additionally, in [40], Qing Wu et al. proposed a path planning algorithm based on the beetle antennae search (BAS), considering requirements such as the path length, maximum turning angle, and obstacle avoidance. By ignoring optimization along the Z-axis and integrating the minimum threat surface (MTS), they applied the proposed path planning algorithm to UAV path planning. The results show that it significantly shortened the path length and maintained a high success rate in generating excellent paths, even in non-convex scenarios. In [41], Wang et al. combined swarm intelligence algorithms with a feedback-based step-size update strategy in the beetle swarm antenna search (BSAS) algorithm. They search in *k* directions at each iteration to find positions with smaller objective function values while evaluating the possibility of the beetle missing better positions of the objective function. By comparing with random numbers, they decide whether to update the step size. The results demonstrated that BSAS outperforms BAS, showing better performance. In [42], Khan et al. introduced a nature-inspired robust meta-heuristic algorithm. Their algorithm utilizes a novel beetle structure and optimization mechanism, assuming that the beetle has multiple antennae distributed with the same angular spread. Gradient estimation is represented as the difference between the average values of the objective function for two sets of non-overlapping antennae. They employ the adaptive moment estimation (ADAM) update rule to adaptively adjust the step size at each iteration. The results showed a significant improvement in convergence speed compared to the original BAS, with a more accurate gradient estimation model. All of the mentioned algorithms challenge the inherent limitations of the BAS algorithm, and each of them has valuable insights that can be learned and applied in further research.

### 2.3. UAV Target Tracking System

Unmanned aerial vehicles (UAVs) have seen significant advancements in autonomous tracking technology, thanks to the development of sophisticated path planning algorithms and advanced object detection mechanisms. The main challenge of UAV-based target tracking lies in the ability to efficiently plan paths while maintaining real-time performance, especially when dealing with complex environmental constraints and dynamic targets. Over the years, researchers have proposed various methods to optimize these tracking systems. This review highlights key developments and methodologies in UAV target tracking, focusing on path planning, object detection, and tracking algorithms.

One of the primary challenges in UAV target tracking is optimizing path planning algorithms to strike a balance between path length, computational complexity, and convergence speed. Efficient path planning must ensure that the UAV follows short paths while keeping computational overhead low. This is crucial for ensuring real-time responsiveness, especially when UAVs need to process large-scale environmental data. To address these challenges, Han [43] proposed an aerial tracking framework that uses polynomial regression to predict target motion based on past observations. The trajectories generated from this method are extrapolated to predict future movements of the target, allowing the UAV to quickly adjust its position. However, the reliance on AprilTag for target detection restricts its applicability, and the system’s inability to perform simultaneous target and environmental perception effectively with limited onboard vision highlights a key limitation. The challenge of maintaining real-time performance while addressing complex environmental factors remains a critical concern for UAV-based tracking systems.

With the rise of deep learning technologies, more advanced techniques for object detection and tracking have been introduced into UAV systems. Pan [7] developed a lightweight human-body detection method using deep learning and nonlinear regression to enhance the UAV’s ability to detect and localize targets. They further improved the UAV’s tracking module by incorporating an occlusion-aware mechanism, allowing the UAV to anticipate potential obstructions and plan accordingly. These improvements enabled a more robust and flexible tracking system. Despite these advances, the system faced challenges when attempting to balance the multiple constraints needed for effective path planning. Ensuring that trajectories meet criteria like safety, visibility, smoothness, robustness, and efficiency in real-time target tracking can be difficult, especially when these properties conflict with each other in certain scenarios. The inconsistency among these constraints necessitates adaptive capabilities within the constraint-solving algorithm to maintain high performance.

To address these multi-faceted challenges, ji [8] introduced the elastic tracker, which presents a more adaptive solution for UAV target tracking. The elastic tracker utilizes an occlusion-aware path search mechanism and introduces the concept of the intelligent safe flight corridor (SFC). By evaluating occlusion costs and developing a specialized mechanism to avoid obstructions, the system improves tracking accuracy, maintaining an appropriate observation distance between the UAV and its target. However, one of the key drawbacks of this system is its dependence on the A* algorithm [44,45] for the hierarchical multi-target path search. While the A* algorithm is reliable, it is relatively outdated compared to more modern algorithms, which could limit the system’s performance in dynamic and complex environments. Additionally, while the use of OpenPose [46] offers lightweight human body detection, its applicability to other entity categories remains limited, reducing the system’s flexibility.

While existing UAV tracking systems have made significant strides in addressing challenges like path planning and object detection, there is still room for further improvements. For instance, real-time UAV tracking systems must continue to optimize computational efficiency, especially in embedded systems with limited processing power. Balancing the demand for fast, real-time decision-making with the need for robust and accurate tracking of multiple dynamic targets under varying environmental conditions remains a challenge. Moreover, the integration of more sophisticated detection methods, such as multi-modal data fusion, could further enhance the adaptability and accuracy of UAV systems in tracking various types of targets. As UAV technology continues to evolve, future research should focus on improving algorithmic adaptability to diverse scenarios and enhancing the robustness of UAV systems in more extreme environmental conditions.

In summary, UAV target tracking systems have evolved significantly with advances in path planning and deep learning-based object detection. However, despite numerous proposed frameworks and methodologies, balancing real-time computational demands with robustness and adaptability in dynamic environments remains a challenge. While solutions such as polynomial regression-based tracking, occlusion-aware mechanisms, and elastic trackers offer promising results, further work is required to refine these systems for broader applications and to address their current limitations.

## 3. Methodology

The overall mission of this plan is to help the drone find a feasible path to reach the target and complete the tracking task. For the tracking scenarios, we make the following reasonable assumptions during the simulation phase:

(1) It is assumed that the displacement of the target in the Z-axis direction is negligible during its movement.
(2) The initial positions and velocities of the UAV and the targets are both assumed to be known and within a certain range.

In this study, it is assumed that the displacement of the target in the Z-axis direction is negligible during its movement. This assumption is grounded in typical UAV target tracking scenarios where targets, such as vehicles or pedestrians, predominantly move within a two-dimensional plane, making vertical movement minimal. Therefore, focusing on the X- and Y-axes simplifies the tracking process without significantly impacting the overall performance. However, for scenarios involving substantial Z-axis displacement, such as aerial or climbing targets, this assumption would need to be reconsidered. In future work, incorporating depth sensors or 3D tracking algorithms could address this limitation and improve tracking performance in such contexts.

### 3.1. Front-End Optimization

#### 3.1.1. Preliminaries of Bionic Algorithm

**Figure 2:**
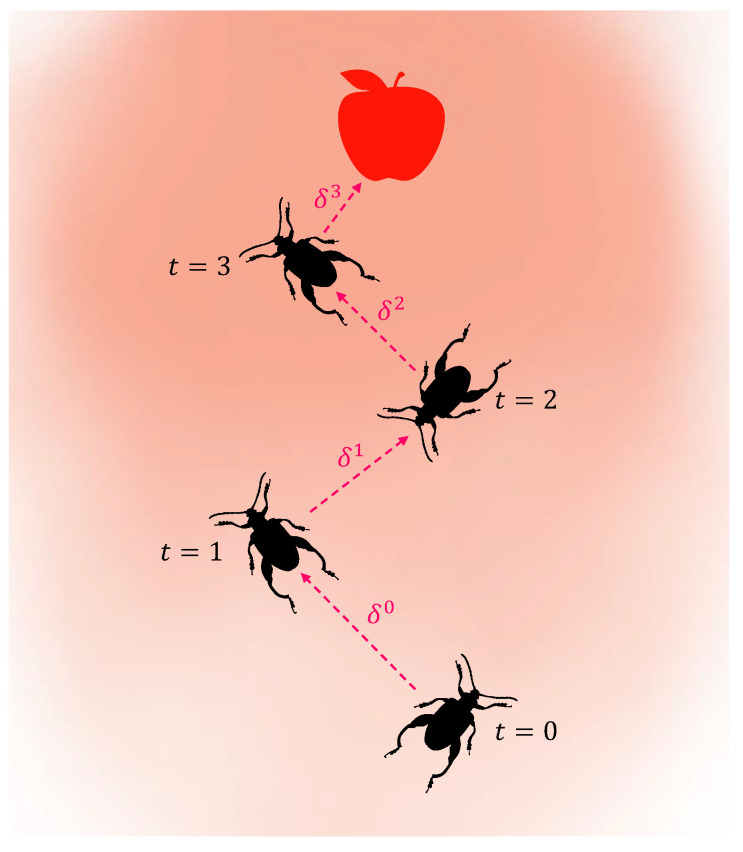
The bionic working principle, with the intensity of color being the odor distribution.

In the BAS algorithm, the foraging behavior of beetles is modeled, and before each movement, a random unit vector $\vec{b}\in R^{n}$ is used to represent the direction. Beetles rely on antennae on both sides of their bodies to sense the odor of food and choose the direction with the stronger food odor to move. They continue searching for food using this mechanism until they find it. In the BAS algorithm, the antennae are fixed on both sides of the beetle, and the coordinates of the left and right antennae of the beetle are represented as

(1) $$\left\{\begin{array}{c} x_{l}=x^{t}-d^{t}\vec{b},\\ x_{r}=x^{t}+d^{t}\vec{b}, \end{array}\right.$$

where the position of $x_{l}\in R^{n}$ represents the location of the left antenna of the beetle, while $x_{r}\in R^{n}$ represents the location of the right antenna of the beetle. In addition, *d* represents the distance from the antennae to the centroid, and $\vec{b}\in R^{n}$ is used to represent the direction. Specifically, $d^{t}\in R^{1}$ represents the search range of the beetle, which decreases over time, as described in Equation (3). The value of $d_{0}$ ensures that $d_{t}$ does not decrease to zero. It is worth mentioning that the magnitude of $d_{0}$ depends on the scale of the specific problem. Once $x_{l}$ and $x_{r}$ are obtained, the algorithm uses a heuristic function f(x) to evaluate the quality of the two points and selects the smaller one as the candidate point. The foraging behavior of the beetle is described by Equation (2).

(2) $$x^{t}=x^{t-1}-\delta^{t}\vec{b}\mathrm{sign}\left(f\left(x^{l}\right)-f\left(x^{r}\right)\right),$$

where the symbol $\delta^{t}$ represents the step size, which decreases over time, as indicated in Equation (4). The update rules for d and δ are described in Equations (3) and (4) as follows:

(3) $$d^{t}=\eta_{d}d^{t-1}+d_{0},$$

(4) $$\delta^{t}=\eta_{\delta }\delta^{t-1}+\delta_{0},$$

where the symbols $\eta_{d}$ and $\eta_{\delta }$ represent the decay coefficients for the antennae length *d* and the step size δ, respectively. Jiang and Li [47] pointed out that the predefined values of parameters (e.g., *d* and δ) have a significant impact on the performance of the BAS. A schematic diagram of the BAS algorithm is shown in Figure 2. After each movement, the beetle randomly changes its orientation, and its position is updated according to Equation (2). The red dashed lines with arrows indicate the direction of movement. It is evident that the length of the red dashed line representing the step size gradually decreases over time, following Equation (4). It is important to note that the process depicted in Figure 2 does not include any additional constraints.

#### 3.1.2. Grid-Based Beetle Antennae Search

The BAS algorithm faces two inherent problems in its application to path planning. Firstly, due to the random orientation of the beetles, it cannot guarantee that the beetle’s movement will lead to a decrease in the objective function. This can result in generated paths that may not meet our expectations or even fail to converge. Therefore, the algorithm uses a quadratic search mechanism to enable the beetle to search for grids with lower costs more efficiently. The search step length $S^{t}$ can be defined as follows:

(5) $$S^{t}=\left\lceil \delta^{t}\right\rceil .$$

Before each beetle searches for the next position, we define a square with the current position of the beetle as the centroid and $2S^{t}$ as the side length. The grids on this square form the search area, which we represent using a set of mappings M(·) here. As shown in Equation (5), the search step length $S^{t}$ is determined by taking the ceiling of the step size $\delta^{t}$, representing the beetle’s search step length. The side length of a square is defined as twice the search step length $2S^{t}$, with the beetle’s current position at the center of this square. This square is then overlaid on a grid map, ensuring that the center of the square coincides with the beetle’s current position. Consequently, the number of grid cells touched by this square is exactly $8S^{t}$, which represents the search range for the beetle in the current iteration. The range of $\left[1,8S^{t}\right]$ ensures that the beetle searches within a well-defined area, facilitating effective exploration without excessive overlap and thus maintaining computational efficiency. We randomly select an integer ϵ from the range $\left[1,8S^{t}\right]$ and use ϵ as a random index instead of a random direction as in the BAS. The left and right antennae of the beetle are now represented as

(6) $$\left\{\begin{array}{c} n_{l}^{t}=M\left(\epsilon \right),\\ n_{r}^{t}=M\left(\mathrm{mod}\left(\epsilon +4S^{t},8S^{t}\right)\right). \end{array}\right.$$

After the beetle relies on its left and right antennae to find a grid with a smaller objective function, it does not move immediately. Instead, it adjusts its orientation to face that grid. The value of ϵ is updated to the index of the grid toward which the beetle is oriented. The beetle then turns its left and right antennae forward, and the grid where the antennae are located is represented as

(7) $$\left\{\begin{array}{c} c_{l}^{t}=M\left(\mathrm{mod}\left(\epsilon -S^{t},8S^{t}\right)\right),\\ c_{r}^{t}=M\left(\mathrm{mod}\left(\epsilon +S^{t},8S^{t}\right)\right). \end{array}\right.$$

**Figure 3:**
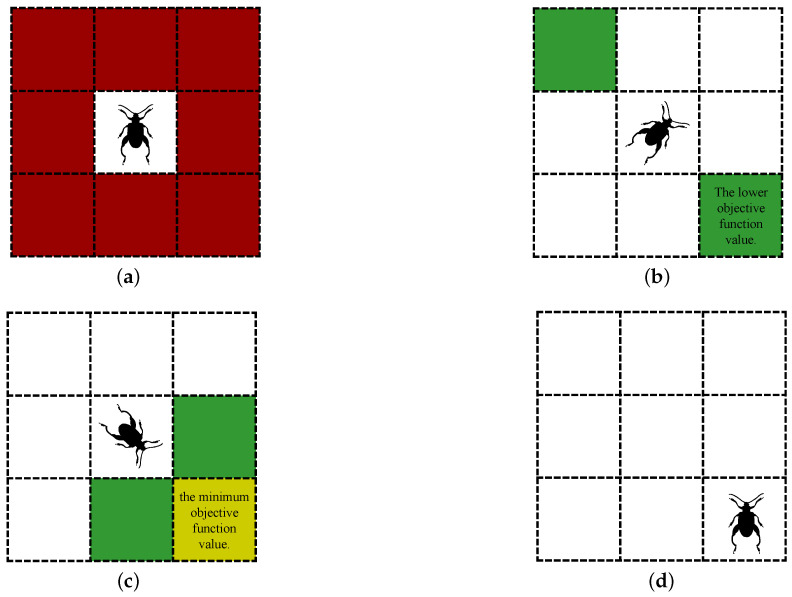
Illustrates one iteration of the GBAS algorithm under the assumption of a step size of 1. (**a**) The red region represents the search space of the beetle. (**b**) The beetle randomly orients itself toward a grid, and the green region represents the grid where the beetle’s antennae are located. (**c**) The beetle reorients itself toward a grid with a lower objective function value and adds it to the candidate list. Then, the beetle tilts its antennae forward and adds the grid where the antennae are located to the candidate list as well. (**d**) The beetle moves to the grid with the minimum objective function value.

At this time, the beetle has three candidates: M(ϵ), $c_{l}^{t}$, and $c_{r}^{t}$. The beetle then removes unavailable nodes from the candidates. This includes Discarded nodes and Occluded nodes during algorithm iterations. Discarded nodes refer to the set of nodes where the beetle was before the rollback mechanism started. Occluded nodes refer to nodes that the detected beetle cannot reach in a straight line from its current location. The beetle uses this to escape the local extreme areas and ensure the safety of the search. Then, the node with the smallest cost function value is selected among the remaining candidate nodes as the destination. Therefore, the secondary search mechanism of the GBAS is shown in Figure 3. Under the conditions of search step size $s^{t}=1$ and no unavailable nodes, Figure 3a shows the search range of the beetle. Figure 3b shows the beetle’s first search. Figure 3c shows the beetle’s second search. Figure 3d shows the beetle moving to its destination.

Secondly, in the BAS, the beetle’s step length decreases over time to ensure the effective convergence of the algorithm. However, when the objective function shows good improvement, it is unnecessary to reduce the step length. Instead, we only need to reduce the step length when the beetle is about to reach its destination to ensure the convergence of the algorithm. Therefore, we have designed a step-length update strategy based on feedback distance, as shown below:

(8) $$\delta^{t}\left(D^{t}\right)=\left\{\begin{array}{cc} \frac{1}{1+e^{k_{1}\left(r-D^{t}\right)}}\delta_{0}, & \mathrm{if}\;D^{t}>r,\\ k_{2}\left[{\left(D^{t}\right)}^{3}-{\left(r\right)}^{3}\right]+\frac{1}{2}\delta_{0}, & \mathrm{if}\;0<D^{t}<r. \end{array}\right.$$

**Figure 4:**
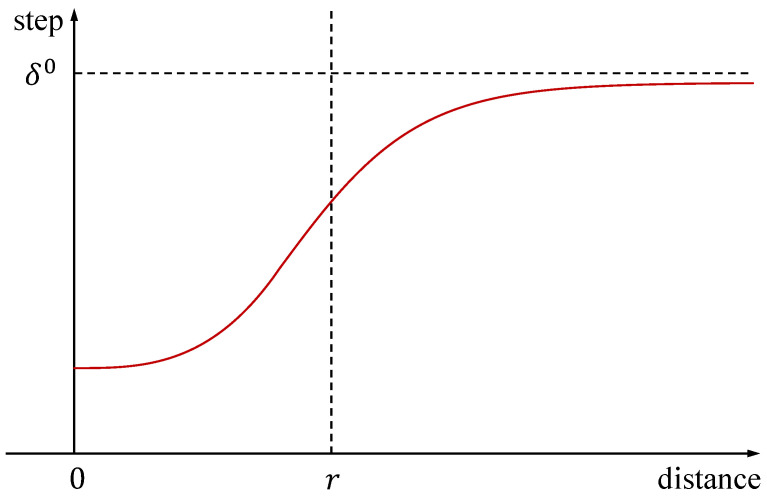
In order to achieve convergence within the desired distance, we have designed a step-size update based on distance feedback between the drone and the target. When the distance is greater than the desired distance, the step size decreases slowly. When the distance is less than the desired distance, the step size decreases rapidly.

The variable $D_{t}$ represents the distance between the current position of the beetle and the target. The variable *r* represents the desired convergence distance. Both $k_{1}$ and $k_{2}$ are small constants. When the distance is greater than the desired distance, the step size decreases slowly. When the distance is less than the desired distance, the step size decreases rapidly, as shown in Figure 4. Based on the characteristics of the GBAS, the cost function is designed as follows:

(9) $$f^{t}\left(n\right)=\sqrt{{\left(d_{xy}^{t}\left(n\right)-d_{d}\right)}^{2}}+\sqrt{d_{z}^{t}{\left(n\right)}^{2}}+\alpha b^{t}O^{t}.$$

**Figure 5:**
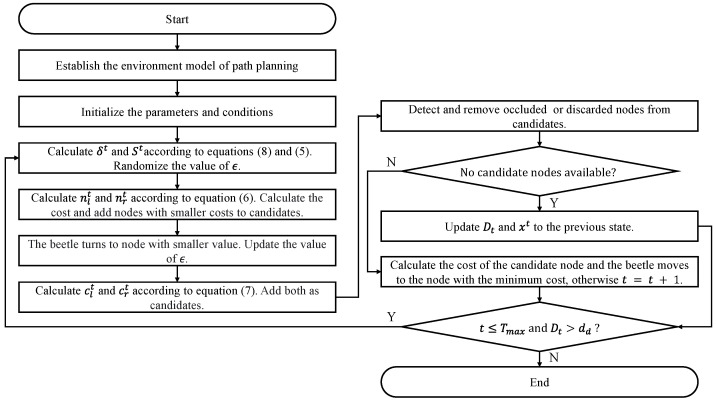
Flowchart of proposed GBAS algorithm.

In the given expression, $d_{xy}^{t}$ represents the distance between node *n* and the target in the X-plane and Y-plane. In addition, $d_{d}$ is a constant representing the desired tracking distance, $d_{z}^{t}$ represents the distance between node *n* and the target along the Z-axis, α is a constant coefficient, $b^{t}$ represents the vector pointing from the current position of the beetle to the target, and $O^{t}$ represents the vector pointing from the current position of the beetle to node *n*. Therefore, we have designed a GBAS enhancement algorithm, as shown in Algorithm 1. In addition, the GBAS flowchart is shown in Figure 5; the parameter list of the GBAS is shown in Table 1.
**Algorithm 1** GBAS algorithm for path planning.**Input:** Given the target position $z_{0}$ and the current position of the UAV $x_{0}$. Establish the cost function $f^{t}\left(n\right)$. The variables *n*, $\delta_{0}$, $\delta_{t}$, $S^{t}$, ϵ, *M*, $D_{t}$, *r*, and $d_{d}$ are initialized.**Output:** The list of waypoints $X={\left[x^{0},\ldots ,x^{n}\right]}^{T}$.  1: **while** 
$t<T_{\max}\;\mathrm{and}\;D_{t}>d_{d}$ **do**  2:    Calculate $\delta_{t}$ and $S^{t}$ according to Equations (8) and (5).  3:    Update the mapping *M*.  4:    $\epsilon =\mathrm{random}(1,8S^{t})$.  5:    **if** $f^{t}\left(M\left(\epsilon \right)\right)>f^{t}\left(M\left(\mathrm{mod}\left(\epsilon +4S^{t},8S^{t}\right)\right)\right)$ **then**  6:       $\epsilon =\mathrm{mod}(\epsilon +4S^{t},8S^{t})$.  7:    **end if**  8:    Calculate $c_{l}^{t}$ and $c_{r}^{t}$ according to Equation (7).  9:    Detect and remove Occluded or Discarded nodes.10:    **if** No candidate nodes available **then**11:      Update $D_{t}$ and $x^{t}$ to the previous state.12:      Continue.13:    **end if**14:    $x^{t}=\mathrm{argmin}\left\{f^{t}\left(c_{l}^{t}\right),f^{t}\left(c_{r}^{t}\right),f^{t}\left(M\left(\epsilon \right)\right)\right\}$.15:    $X=[X,x^{t}]$.16: **end while**17: **return**
*X*

The authors would like to clarify that the grid-based beetle antennae search (GBAS) algorithm, as a path search algorithm, is designed to generate safe and efficient paths without directly considering the aircraft’s dynamics. The role of the GBAS is to ensure that an optimal and feasible trajectory is planned.

However, the aircraft’s dynamic model, including the turn radius and other motion constraints, is addressed in a subsequent path optimization step. For path optimization, we followed previous work [8]. This step ensures that the generated path can be followed by the UAV in accordance with its physical limitations.

#### 3.1.3. Time Complexity Analysis

Time complexity is a method of measuring the merits and complexity of the gradient algorithm and is also a tool to reflect the performance of the algorithm [48,49]. The main determinants of time complexity are the spatial dimensions of the model and the objective function.

The GBAS algorithm consists of a main loop that iterates until a maximum number of iterations $T_{\max}$ is reached or the distance to the goal is less than a desired distance $d_{d}$. The main loop of the GBAS algorithm mainly includes the step-size update, the generation of the search range $M_{r}$, the calculation cost, and node elimination. The step-size update is completed in constant time, so its time complexity is O(1). Generating the search range $M_{r}$ means generating $8S_{t}$ nodes around the current position, so its time complexity is $O(S_{t})$. The calculation cost is also completed in constant time, so its time complexity is also O(1). Node elimination checks whether a node is out of bounds or occluded and involves traversing a set of nodes $n_{\mathrm{occ}}$ and performing checks. The time complexity is $O(n_{\mathrm{occ}})$, with $n_{\mathrm{occ}}$ being a constant.

Therefore, assuming that the number of iterations used by the GBAS is *t*, the time complexity of the GBAS is depicted as follows:

(10) $$T=O\left(t\left(1+S_{t}+1+n_{\mathrm{occ}}\right)\right)=O\left(tS_{t}\right).$$

In general, the time complexity of the GBAS algorithm is mainly consumed in the search range generation.

### 3.2. Confusion-Aware Object Localization

#### 3.2.1. Multi-Class Object Detection

Considering real-time performance and accuracy, we applied YOLOv8 [50]. The You Only Look Once version 8 (YOLOv8) is a significant technological advancement in the field of object detection. It integrates the strengths of the YOLO series and introduces further innovations. Its distinguishing feature lies in combining efficient real-time detection with accurate localization capabilities, achieving object detection in a single forward pass. This not only results in high speed but also maintains a high level of precision.

YOLOv8 achieves improved object classification and position prediction by incorporating a more powerful neural network architecture and enhanced training strategies. This enables it to better handle challenges such as detecting small-sized objects and densely arranged objects in complex scenes. Furthermore, YOLOv8 has been optimized in areas like data augmentation and model fusion, enhancing the model’s generalization capabilities.

For multi-class object detection, we tested various object detection networks, and the results are presented in Section 4.4.

#### 3.2.2. Confusion-Aware Mechanism

In a real-world scenario, relying solely on target detection to obtain the position information for a UAV tracking system presents issues with insufficient robustness. In particular, when the UAV’s field of view includes similar target types, target detection may generate multiple target bounding boxes, posing challenges of perceptual confusion in the UAV tracking process. To overcome this issue, further measures can be taken to enhance the accuracy and reliability of tracking.

One strategy is to introduce target feature recognition. Assuming stable environmental lighting during UAV tracking, given that targets can change in scale and that real-time performance demands are high, we choose to implement target recognition using oriented FAST and rotated BRIEF (ORB) [51] feature points. After target detection, we extract ORB feature points from the targets and establish a feature point dictionary. Through feature point matching, we can identify the targets. This approach distinguishes targets in the field of view with similar targets by utilizing the uniqueness and stability of feature points, thus enhancing tracking accuracy.

Another strategy is to combine target tracking algorithms with the initial position information provided by target detection. During tracking, we can use the initial position information provided by the target detection algorithm as the starting point for tracking and then employ motion estimation techniques to continuously track the targets in subsequent frames. This strategy yields continuous target trajectories, overcoming potential issues from multiple bounding boxes introduced by target detection and enhancing the robustness and accuracy of tracking.

To address the challenge of perceptual confusion due to the presence of similar targets within the UAV’s field of view, we adopt the approach of ORB feature point extraction and dictionary matching to correctly identify and track the targets. The ORB algorithm possesses characteristics such as rotational invariance, scale invariance, and high computational speed, making it well suited for real-time visual systems.

In this mechanism, we first employ the target detection algorithm to detect the objects within the input image, obtaining their positions and bounding box information. Then, for each bounding box region, we extract the ORB feature point set ${\mathrm{KP}}^{k}$ and generate a descriptor set $D^{k}$:

(11) $${\mathrm{KP}}^{k}=\left\{{\mathrm{kp}}_{1}^{k},{\mathrm{kp}}_{2}^{k},\ldots ,{\mathrm{kp}}_{n}^{k}\right\},$$

(12) $$D^{k}=\left\{{\mathrm{des}}_{1}^{k},{\mathrm{des}}_{2}^{k},\ldots ,{\mathrm{des}}_{n}^{k}\right\}.$$

In the above expression, ${\mathrm{kp}}_{n}^{k}$ represents the pixel coordinate of the n-th feature point in the k-th bounding box. ${\mathrm{des}}_{n}^{k}$ represents the descriptor of the n-th feature point in the k-th bounding box. ORB uses the BRIEF algorithm to calculate the descriptor of a feature point. The core idea of the BRIEF algorithm is to select multiple point pairs in a certain pattern around key points and combine the comparison results of these multiple point pairs as descriptors. We gradually construct these trusted descriptors of the tracking target bounding box into a descriptor dictionary Dic as a reference for the target features:

(13) $$\mathrm{Dic}=\mathrm{Dic}\cup \left\{{\mathrm{des}}_{i}^{ref}\right\},\;\;\mathrm{if}\;{\mathrm{des}}_{i}^{ref}\notin \mathrm{Dic},$$

where ${\mathrm{des}}_{i}^{ref}$ represents the descriptor of the *i*-th feature point in the trusted bounding box.

In terms of finding trusted bounding boxes, in subsequent image frames, we also perform target detection and bounding box extraction. Then, we extract the ORB feature points and descriptors within the bounding box regions. We match the descriptors corresponding to the feature points within the bounding boxes of the subsequent image frames with the previously constructed descriptor dictionary Dic. Specifically, for the descriptor ${\mathrm{des}}_{i}^{k}$ corresponding to the i-th feature point in the k-th bounding box $b_{k}^{t}$ in the t-th frame image, if there is a descriptor ${\mathrm{des}}_{j}^{\mathrm{Dic}}$ in the dictionary Dic, and this ${\mathrm{des}}_{i}^{k}$ is calculated through the similarity matching function and the result is greater than the confidence CS, the match is successful. The descriptor ${\mathrm{des}}_{i}^{k}$ is considered trusted. This increases the feature confidence $N_{k}^{t}$ of the current bounding box:

(14) $$N_{k}^{t}=\sum_{i=1}^{m}\left\{\begin{array}{cc} 1, & \mathrm{if}\;\exists j:\mathrm{sim}({\mathrm{des}}_{i}^{k},{\mathrm{des}}_{j}^{\mathrm{Dic}})>CS,\\ 0, & \mathrm{otherwise}. \end{array}\right.$$

where *m* is the total number of detected bounding boxes in the image. The confusion-aware mechanism uses this method to calculate the score of each bounding box. All calculated bounding box scores are combined into a set $NG^{t}$:

(15) $${\mathrm{NG}}^{t}=\left\{N_{1}^{t},N_{2}^{t},\ldots ,N_{k}^{t}\right\},$$

In this case, the confusion-aware module selects the highest-scoring bounding box as the final output bounding box $B^{t}$ of the t-th frame image.

(16) $$B^{t}=\arg\max_{k}NG^{t}.$$

This feature matching algorithm accomplishes target identification and tracking. Poorly matched bounding boxes are considered invalid. To enhance robustness, in situations where multiple targets exist in the field of view, target positioning information is compared to predicted positions, and targets with large positional discrepancies are marked as invalid. This perceptual confusion mechanism offers more stable and accurate target tracking capabilities, particularly suitable for scenarios with substantial variations in target scale and appearance.

**Figure 6:**
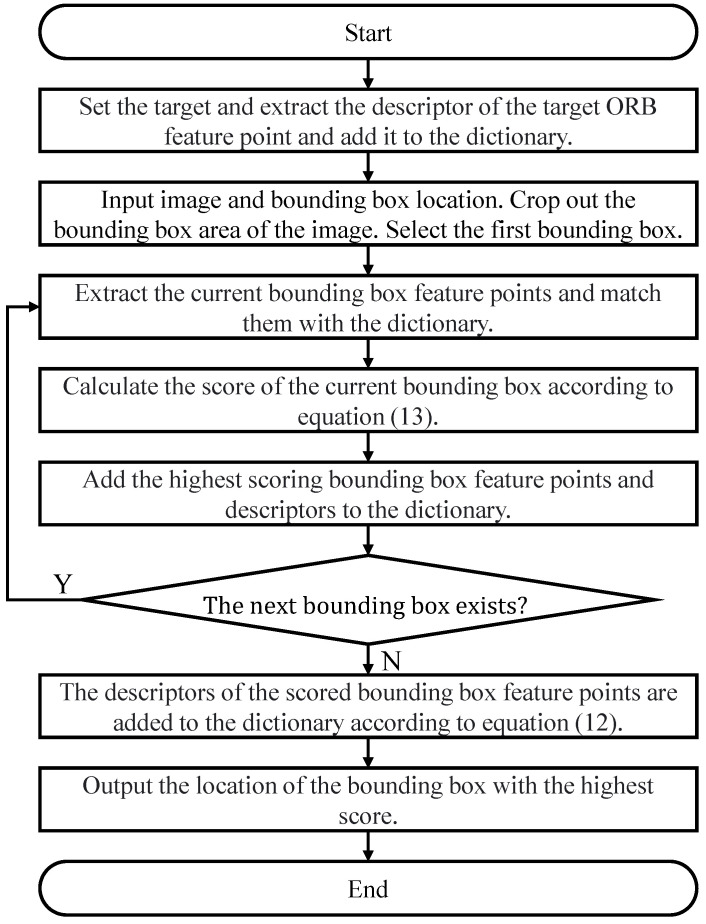
Flowchart of confusion-aware mechanism for distinguishing tracked targets.

By integrating ORB feature point extraction, dictionary construction, matching, and position information comparison, we are able to accurately identify and track targets, mitigating confusion scenarios. This mechanism provides a reliable target tracking solution for real-time UAV visual systems. The flowchart of the confusion-aware mechanism for distinguishing tracked targets is shown in Figure 6.

#### 3.2.3. Depth-Based Object Localization

Regarding object localization, we employ a common depth-based localization method [52]. Firstly, considering the influence of noise and outliers on depth estimation, we use the average depth value $S^{t}$ of multiple feature points within the bounding box obtained from object detection to represent the depth of the target. The depth information is obtained from a depth camera. Secondly, we convert the target pixel coordinates ${\left(u^{t},v^{t}\right)}^{T}$ to normalized coordinates ${\left(x^{t},y^{t},1\right)}^{T}$, as depicted below:

(17) $$\left\{\begin{array}{c} x^{t}=\frac{u^{t}-c_{x}}{f_{x}},\\ y^{t}=\frac{v^{t}-c_{y}}{f_{y}}. \end{array}\right.$$

The coordinates ${\left(c_{x},c_{y}\right)}^{T}$ represent the origin coordinates, which correspond to the position of the camera’s optical center on the image plane. The values ${\left(f_{x},f_{y}\right)}^{T}$ represent the focal lengths. To obtain the coordinates ${\left(x_{c}^{t},y_{c}^{t},z_{c}^{t}\right)}^{T}$ of the target in the camera coordinate system, it can multiply the normalized coordinates by the depth value $S^{t}$, as shown in the following equation:

(18) $$\left\{\begin{array}{c} x_{c}^{t}=x^{t}S^{t},\\ y_{c}^{t}=y^{t}S^{t},\\ z_{c}^{t}=S^{t}. \end{array}\right.$$

**Figure 7:**
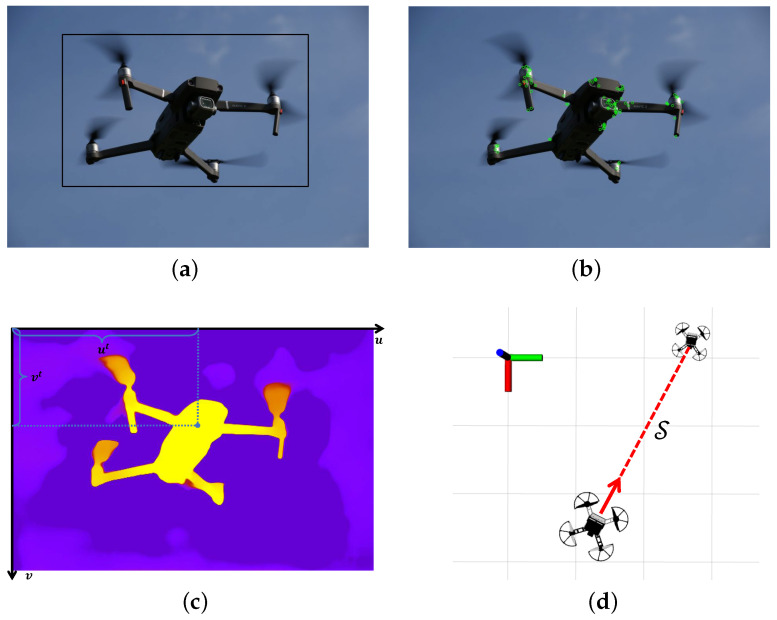
The confusion-aware target localization method workflow. (**a**) Object detection output bounding boxes. (**b**) Feature point extraction and matching. (**c**) Calculating the average depth using a depth map. (**d**) Locating based on the average depth.

From this, the UAV system obtains the coordinates in the coordinate system of the target camera. Therefore, the workflow of confusion-aware object localization is as shown in Figure 7. Figure 7a shows the output bounding box of object detection. In addition, Figure 7b shows the ORB feature points extracted in the confusion-aware mechanism. Figure 7c shows the calculation of the average depth. Figure 7d shows the target location information obtained by the drone.

## 4. Results and Discussion

### 4.1. Parameter-Sensitive Testing

This section presents a performance test of the GBAS algorithm and the chaos perception module. In addition, it also compares the performance of some current mainstream target detectors to optimize the target detector for the tracking system. All experiments were conducted on a computer configured with an Intel i5-12400 CPU, an NVIDIA RTX 3060 12 GB GPU, and 64 GB RAM.

In this section, we validate the performance and convergence of the GBAS in both the Matlab and ROS environments to verify the algorithm’s effectiveness and its application in elastic trackers. In Matlab, we conducted extensive tests on cost using different parameter settings, such as $\delta^{0}$, *r*, $k_{1}$, and $k_{2}$, and starting from various positions. Generally, on a 50×50 grid map, the maximum iteration count for the GBAS algorithm was set as $T_{\max}=100$, and the desired distance as $d_{d}=1$. The predefined parameters for the feedback-based step-size update strategy were set as $\delta^{0}=3$, r=2, $k_{1}=3$, and $k_{2}=0.15$. For clarity, in subsequent discussions, we refer to this set of data as the control group.

**Figure 8:**
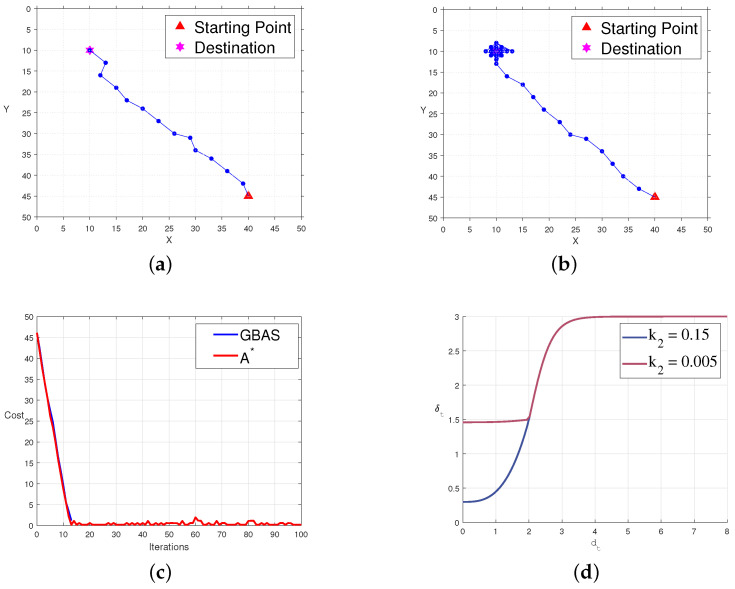
The influence of different parameters on algorithm convergence on an empty map. (**a**) The path of the GBAS when $k_{2}=0.15$. (**b**) The path of the GBAS when $k_{2}=0.005$. (**c**) In the case of different parameters, the decline in the objective function. (**d**) The change curve of the step size with different parameters.

To investigate the influence of predefined parameters on the quality of paths and convergence, we set up comparison experiments with multiple groups of different parameters. We modified the $k_{2}$ parameter for the comparison group, ensuring that the size of the root does not exceed the convergence radius for efficient path convergence. The predefined experimental parameters for the comparison group were $\delta^{0}=3$, r=2, $k_{1}=3$, and $k_{2}=0.005$, referred to as the experimental group. The comparison experiments are shown in Figure 8. In Figure 8a, the path of the control group is displayed in an open environment. In Figure 8b, the path of the experimental group in an open environment is shown. Figure 8c presents the variation in cost functions for the experimental and control groups with iterations, where blue represents the control group, and red represents the experimental group. Figure 8d illustrates the relationship between the step size $\delta^{t}$ and the distance $d_{t}$ to the target for both the experimental and control groups, with blue indicating the control group and red indicating the experimental group. From the experiments, we found that $k_{2}$ primarily affects the size of the root in the step-size update strategy, with smaller $k_{2}$ leading to larger roots. In practical applications, $k_{2}$ should not be too small to ensure quick path convergence. However, setting it too large should be avoided to prevent the agent from losing its ability to act right after entering the convergence area *r*. We also tested parameters like $k_{1}$, *r*, and $\delta^{0}$, and the results indicated that their values all require empirical guidance to align with practical applications.

### 4.2. Number Function Evaluation

The number function evaluation for the optimization algorithm was carried out using the CEC test functions, which incorporate experiments conducted across dimensions of 10, 30, 50, and 100. The CEC test suite consists of 30 truly challenging benchmark problems, including 1–3 single-modal, 4–10 multi-modal, 11–20 hybrid, and 21–30 composite features. Note that the results of F2 are usually abandoned in the CEC test. This dataset is a recent and highly complex one and includes all major types of optimization problems. A general discussion on the definition of these benchmark problems can be found in [53].

In order to adapt the proposed GBAS algorithm for CEC testing, several mechanisms are optimized within the algorithm environment. This includes the integration of a quadratic search and step-size update mechanism. Specifically, the proposed GBAS algorithm performs a quadratic search in each dimension after initial random searches, focusing subsequent efforts on regions with lower fitness values. The step-size function is designed to decrease with experimental error reduction, as defined below:

(19) $$\delta_{t}=\delta_{i}-\left(\delta_{i}-C\right)\left(\frac{1}{1+\exp\left(k(e_{c}-m)\right)}\right),$$

where $\delta_{t}$ denotes the step size of the algorithm, $\delta_{i}$ represents the initial step size, *C* is a constant adjusting the lower limit of the step-size function, *k* reflects the gradient when the step size decreases, *m* adjusts the influence of error, and $e_{c}$ represents the current position’s error in the algorithm.

The parameter settings are based on the requirements of the CEC and the characteristics of the GBAS optimization algorithm. The parameter settings for the algorithm are shown in Table 2. Table 3 presents the experimental results of the proposed GBAS algorithm on the CEC test functions of varying dimensions.

The above refinements enhance the adaptability and performance of the GBAS across the diverse settings of the CEC test functions, as demonstrated in the experimental outcomes. Judging from the experimental results, the GBAS algorithm performs better when dealing with low-dimensional problems, but as the dimension increases, both the error and standard deviation may increase accordingly, especially on specific functions.

### 4.3. Performance Comparisons and Statistical Tests

**Figure 9:**
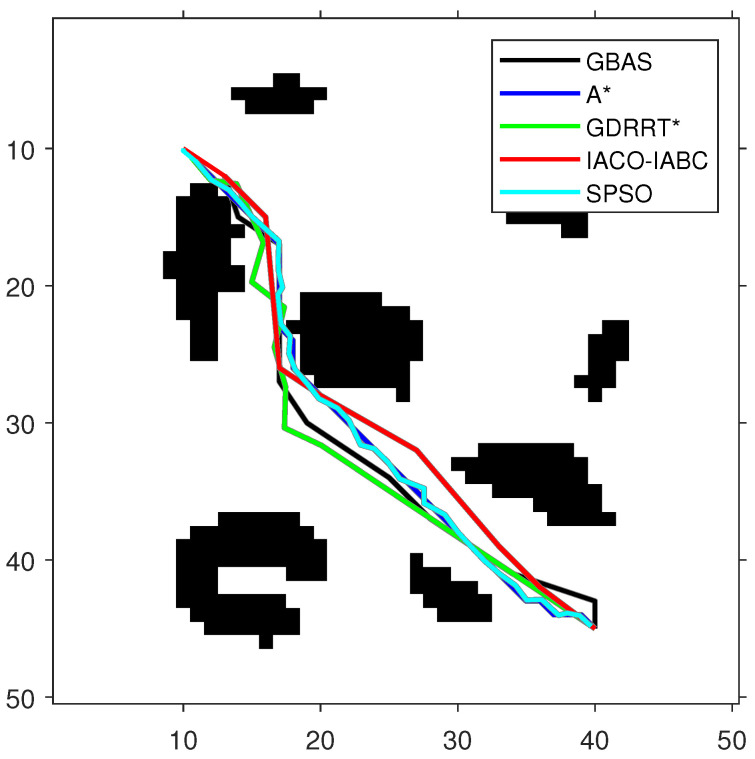
The optimal paths of the tested algorithms in Map 1.

**Figure 10:**
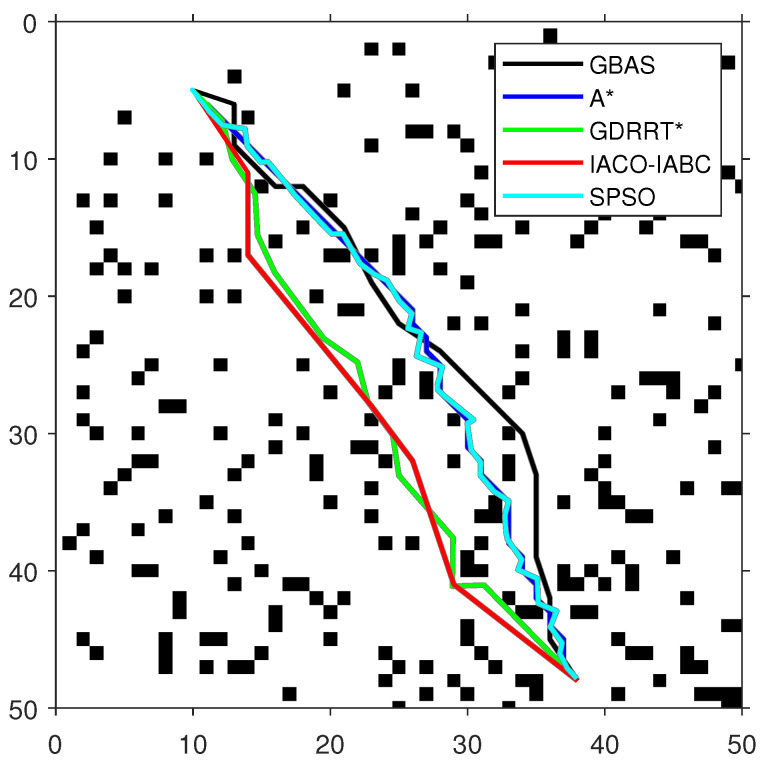
The optimal paths of the tested algorithms in Map 2.

**Figure 11:**
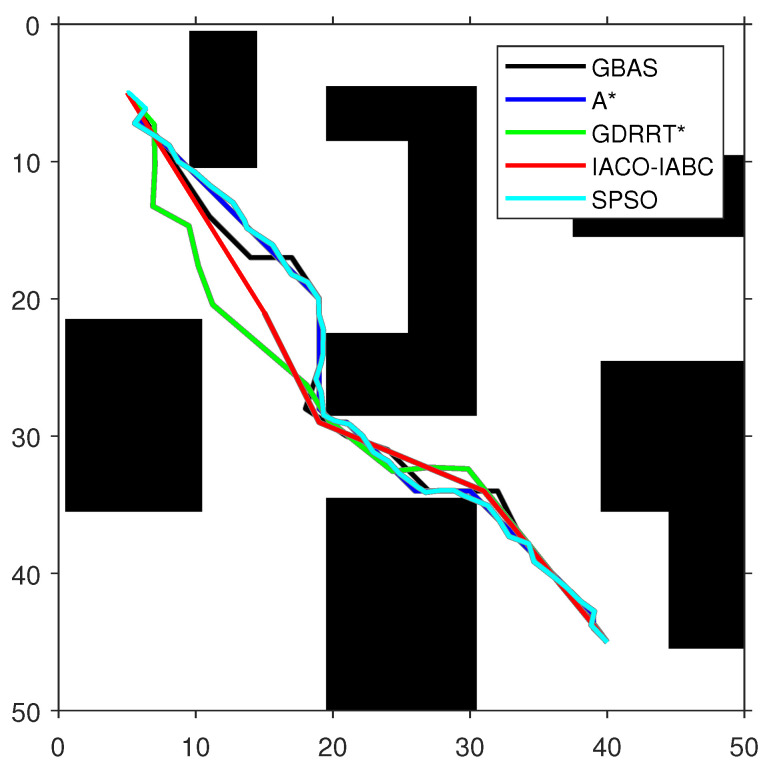
The optimal paths of the tested algorithms in Map 3.

In order to test the performance of the proposed GBAS algorithm, different environmental models were designed in the experiment, as shown in Table 4. On the map, black represents obstacles. The experimental platform is MATLAB. The GBAS algorithm is also compared with other intelligent path planning algorithms. The parameter settings of the GBAS algorithm are as follows: $\delta^{0}=3$, $k_{1}=3$, $k_{2}=0.15$, and r=5. In order to ensure fairness, the general parameters of the GBAS are the same as those of the other compared algorithms; the maximum number of iterations is set to 100. The population number of the population-based methods is consistent, including PSO and IACO-IABC, and their population number is set to SN=20. Methods with range search mechanisms include the GBAS and GDRRT*. Their initial step sizes are the same. The initial step size is set to $\delta^{0}=3$. The special parameters of ACO in the IACO-IABC algorithm are the same as those of the traditional ACO, and the parameters of the ABC algorithm are the same as those of the traditional ABC algorithm. For example, the parameters of ACO include number of ants M=10, pheromone importance α=1, heuristic importance β=7, pheromone evaporation rate ρ=0.3, and pheromone intensification constant Q=1. The parameters of ABC include number of bees N=10, and the maximum number of trials is set to 10. The special parameters of GDRRT* are neighborhood radius $r_{n}=5$, search spherical area diameter d=5, random minimum size $d_{\min}=2$, and minimum obstacle distance $O_{d}=1$. The special parameters of SPSO are inertia weight w=1, cognitive coefficient $\eta_{1}=1.5$, social coefficient $\eta_{2}=1.5$, and number of intermediate points $n_{i}=50$. Then, each algorithm was run 30 times on each map for path planning. The optimal paths obtained by each compared algorithm are shown in Figure 9, Figure 10 and Figure 11, and the experimental results are shown in Table 5. In addition, the speedup depicted in $T_{\mathrm{imp}}$ represents the average running time difference compared to other algorithms, expressed as a percentage, which is positive if there is an improvement:

(20) $$T_{\mathrm{imp}}=\left(T_{\mathrm{com}}-T_{\mathrm{pro}}\right)/T_{\mathrm{pro}}\cdot 100\%,$$

where $T_{\mathrm{com}}$ represents the running time of the other algorithms, and $T_{\mathrm{pro}}$ represents the running time of the proposed GBAS algorithm.

As shown in Figure 9, Figure 10 and Figure 11 and Table 5, the path length of the GBAS algorithm and the path lengths of the other four algorithms were tested through the Wilcoxon rank-sum test. It is shown that, except for SPSO, the path length of the proposed GBAS algorithm and other comparative algorithms are better in different environments. All differences are significant. In addition, the average path length of the GBAS algorithm is shorter than those of the other algorithms except for A*, and the standard deviation is also within an acceptable range. In addition, the running time of the GBAS is the shortest among all environments, and the standard deviation is also better, which shows the good real-time performance of the GBAS. The Wilcoxon rank-sum test shows that there is a significant difference in running time between the GBAS algorithm and the other algorithms in different environments. In Map 1, the running time of the algorithm is shortened by more than 100%, reaching a maximum of 495%. In Map 2’s environment with a large number of discrete obstacles and Map 3 with concave obstacles, the performance is not as good as in Map 1, but what is interesting is that the GBAS algorithm performs well in both Map 2 and Map 3. This is due to the quadratic search mechanism and the distance-based step-size adjustment strategy, which allow the proposed GBAS algorithm to converge quickly.

In order to verify the effectiveness of the improvement, on the ROS platform, we integrated the GBAS algorithm into the elastic tracker and used the benchmark method in Ji et al. [8] to conduct comparative testing in a simulated environment. The experimental map contained a discrete obstacle, the target’s speed was set to 1 m/s and 2 m/s, and the same series of target points were set to drive the tracked target to move. There were 40 target points in total, distributed in different locations on the map. The new system was tested against the elastic tracker as the benchmark. The comparative experimental results are shown in Table 6.

Under the condition of target $v_{\max}=$ 1 m/s, the average search time of the GBAS in the path search phase of the two tracking systems is 0.18 ms, which is 233% faster than that of A*, which is 0.96 ms. Under the condition of target $v_{\max}=$ 2 m/s, the average search time of the GBAS in the path search phase of the two tracking systems is 0.22 ms, which is 555% faster than that of A*, which is 1.41 ms.

The initial positions and velocities of the UAV and targets are crucial for trajectory planning, as they determine the starting conditions for tracking. Defining appropriate ranges for these parameters ensures that the UAV can effectively initiate and maintain tracking. In this study, these ranges were used to simulate various scenarios, influencing the UAV’s response time and the complexity of path planning.

Higher velocities require faster decision-making from the UAV to maintain smooth tracking and avoid overshooting. At high speeds, even minor errors can significantly impact the tracking accuracy. Therefore, velocity was carefully evaluated in our experiments.

In the experiments, the target’s maximum speeds were set to 1 m/s and 2 m/s to test the UAV’s ability to track targets at different speeds, providing a benchmark for its performance in varied scenarios.

### 4.4. Object Detection Tests

**Figure 12:**
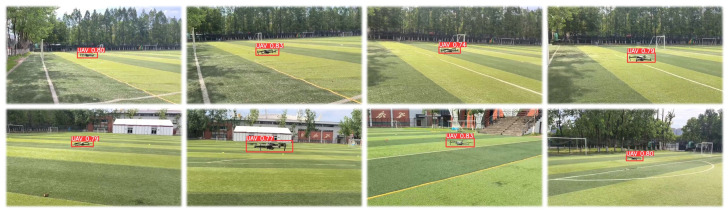
Object detection test set example.

Regarding the object detection dataset, we chose the Det-Fly dataset [54] as the foundation for our training. This dataset is renowned for its systematic and comprehensive nature, encompassing various factors, such as diverse background scenarios, different shooting perspectives, variations in object distances, flight altitudes, and lighting conditions. This makes it an ideal dataset, particularly suitable for training object detection models focused on identifying drones. We selected multiple object detection networks from the Det-Fly dataset for training, including some popular real-time object detectors and end-to-end object detection models. To assess the performance of these models, we constructed a test dataset, as shown in Figure 12, comprising 3194 images, all of which were captured in real-world drone tracking scenarios.

The test results on real-time performance and detection accuracy are presented in Table 7 and Table 8. Table 7 provides insight into real-time performance, showcasing YOLOv8’s ability to balance high FPS and a manageable parameter size. It highlights that the proposed model achieves higher FPS (62 FPS) while keeping parameters (43 M) relatively low compared to similar models, demonstrating efficiency in computation. Table 8 further compares the detection performance and computational cost of different models, where the FLOPs (floating point operations per second) serve as an indicator of computational complexity. Although YOLOv8 (ours) has slightly higher GFLOPs (164 GFLOPs) than other YOLO versions, it compensates with better precision, recall, and F1-score values (85.2%, 92.7%, and 0.89, respectively), indicating that the additional computational complexity leads to superior detection accuracy. Therefore, while YOLOv8’s computational complexity is slightly higher, the trade-off in terms of improved real-time performance and enhanced detection capabilities justifies its inclusion. Among various tested networks, YOLOv8 developed in our model exhibited exceptional performance, making it the chosen object detector for the UAV system.

**Figure 13:**
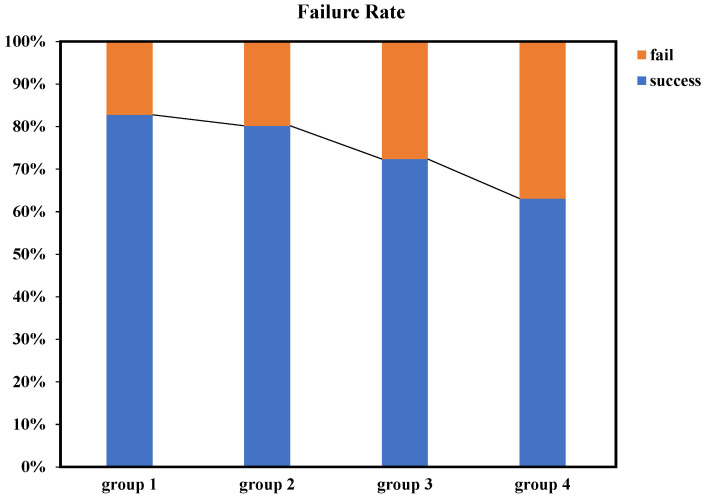
Confusion-aware failure rates in different scenarios: the first group represents experimental sets with varying colors and shapes, the second group consists of experimental sets with different colors but the same shape, the third group includes experimental sets with the same color but varying shapes, and the fourth group comprises experimental sets with both the same color and shape.

In addition to object detection, we conducted a series of tests on the confusion-aware module. To maintain real-time performance, we limited the number of feature points extracted per image to a maximum of 50 and set the confidence threshold for descriptors to 50. For benchmark testing, we selected drone images with different shapes and colors as experimental subjects, ensuring clear distinctions between drones. Furthermore, we selected drone images with the same shape but different colors, as well as drone images with different shapes but similar colors for experimentation. Ultimately, we also tested drones of the same model, with all test results outlined in Figure 13.

The experimental results indicated that the confusion-aware module achieved a high success rate when dealing with significant differences in color or shape features. Even in cases where the differences in color and shape features were less pronounced, the module maintained a reasonable success rate. Apart from success rate testing, we also evaluated the performance of the confusion-awareness. It is important to note that when the object detector failed to detect additional objects, the confusion-aware module remained in a dormant state. During continuous operation, the average processing time of the confusion-aware module was 40 milliseconds. The average computation time for subsequent target localization remained below 6 milliseconds, which is sufficient to meet real-time requirements.

### 4.5. Managerial Implications and Applications

The UAV target tracking system developed in this work holds significant managerial implications, particularly in sectors where real-time monitoring, decision-making, and automation are crucial. For industries like logistics, surveillance, and agriculture, efficient target tracking can reduce human intervention, improve operational safety, and enhance productivity. For example, in logistics, UAVs equipped with advanced tracking systems can autonomously follow delivery routes, detect obstacles, and ensure the timely delivery of packages. Similarly, in surveillance, the system enables UAVs to continuously monitor large areas with minimal human oversight, providing real-time information critical for security operations. These improvements in automation can lead to reduced labor costs and faster response times, thereby increasing overall operational efficiency.

Furthermore, this system’s adaptability to different environmental conditions and its robustness in tracking multiple targets simultaneously make it ideal for dynamic environments where conditions can change rapidly. This capability is particularly beneficial for industries like search-and-rescue operations or agricultural monitoring, where UAVs need to swiftly identify and track moving objects (e.g., humans in distress, wildlife, or livestock) and provide critical data to operators in real time. The integration of a confusion-aware mechanism in the tracking system ensures that UAVs can distinguish between similar targets, thus avoiding errors that could lead to operational inefficiencies or missed objectives.

Beyond UAV tracking, the technologies and algorithms proposed in this work are applicable to other domains where real-time object detection and decision-making are essential. For instance, in autonomous driving, vehicles equipped with similar tracking algorithms could improve their ability to detect and respond to pedestrians or other vehicles in complex urban environments. Similarly, in smart surveillance systems, such algorithms could enhance real-time threat detection, providing quicker alerts and responses in situations requiring immediate attention. Additionally, the framework’s adaptability to handle dynamic, multi-target tracking scenarios positions it as a valuable tool for industrial automation, where it could be applied to track machinery or goods on factory floors, improving workflow efficiency and safety.

## 5. Conclusions

In this paper, the elastic tracker has been enhanced by integrating a novel GBAS algorithm, which improves path efficiency and convergence speed. The addition of a backtracking mechanism further allows the algorithm to avoid local minima, leading to more optimal path planning. The proposed lightweight multi-object detection and localization method, which incorporates a confusion-aware mechanism and depth information, can efficiently handle multiple targets with minimal cost. In particular, the proposed GBAS algorithm has successfully enhanced the elastic tracker by improving path efficiency and convergence speed, as demonstrated through extensive experimental results and statistical tests. The inclusion of the backtracking mechanism also allows the algorithm to avoid local minima, fulfilling the objective of optimizing path planning in complex environments. Furthermore, the confusion-aware multi-object detection and localization method meets the objective of efficiently handling multiple targets with minimal computational cost while still maintaining real-time performance. These contributions, validated through comparative experiments, address both theoretical and practical aspects of UAV target tracking. These advancements contribute significantly to the theory and practice of UAV target tracking, enhancing both performance and reliability. Future work could focus on the compression approach of the algorithm and model and the realization of a lightweight embedded UAV tracking and detection system.

## Figures and Tables

**Table 1:** GBAS algorithm parameters and descriptions.

Parameter	Description
$\delta_{0}$	Initial movement distance.
$k_{1}$	Decay rate as beetle nears target.
$k_{2}$	Adjusts step size based on distance.
*r*	Area within which search stops.

**Table 2:** Parameter settings for the CEC tests.

Parameters	Value	Description
$i_{\mathrm{Max}}$	2000*dim	The maximum number of iterations of the algorithm
ε	1 $\times \;10^{-8}$	The convergence error of the algorithm
$\delta_{i}$	10	The initial step size
*C*	−6	A constant used to adjust the lower limit of the step size
*k*	0.0001	Reflects the gradient when the step size decreases
*m*	5000	The role of adjustment error effects
*L*	5	The maximum number of times without significant improvement
α	1.5	The step-size growth rate
$P_{t}$	1	The total group number

**Table 3:** Number function evaluation results on varying dimensions via proposed GBAS algorithm.

Dimension	10		30		50		100	
**Function**	**Mean**	**Std**	**Mean**	**Std**	**Mean**	**Std**	**Mean**	**Std**
F1	4.07e+03	3.46e+03	5.27e+03	5.64e+03	9.36e+03	9.98e+03	1.61e+04	1.88e+04
F3	0.00e+00	0.00e+00	1.19e+05	3.78e+04	2.32e+05	5.25e+04	6.19e+05	1.12e+05
F4	9.49e+00	1.57e+01	4.25e+01	3.00e+01	5.90e+01	3.80e+01	1.07e+02	4.87e+01
F5	2.47e+02	1.20e+02	7.56e+02	1.57e+02	1.08e+03	1.87e+02	2.29e+03	2.89e+02
F6	9.36e+01	2.06e+01	9.81e+01	1.30e+01	9.80e+01	1.09e+01	9.70e+01	7.53e+00
F7	9.37e+02	2.79e+02	3.85e+03	8.03e+02	7.09e+03	9.33e+02	1.42e+04	1.10e+03
F8	1.61e+02	6.82e+01	6.15e+02	1.65e+02	1.09e+03	1.75e+02	2.48e+03	3.23e+02
F9	3.61e+03	1.45e+03	1.36e+04	2.90e+03	3.00e+04	6.01e+03	5.90e+04	7.06e+03
F10	1.99e+03	3.92e+02	5.63e+03	6.36e+02	8.96e+03	9.54e+02	1.74e+04	1.33e+03
F11	9.45e+01	5.19e+01	1.95e+02	6.31e+01	2.89e+02	6.64e+01	3.30e+04	1.71e+04
F12	4.47e+06	3.66e+06	3.12e+06	1.95e+06	9.59e+06	4.22e+06	2.88e+07	1.44e+07
F13	1.63e+04	1.16e+04	1.66e+04	2.05e+04	1.21e+04	1.07e+04	1.43e+04	7.52e+03
F14	1.06e+04	9.63e+03	6.84e+05	6.14e+05	8.71e+05	4.48e+05	1.24e+06	5.82e+05
F15	6.78e+03	8.01e+03	7.25e+03	1.00e+04	1.00e+04	7.28e+03	3.76e+03	3.67e+03
F16	6.86e+02	2.49e+02	1.67e+03	4.03e+02	2.74e+03	4.97e+02	5.06e+03	7.41e+02
F17	5.17e+02	1.84e+02	1.17e+03	3.78e+02	2.50e+03	4.70e+02	4.65e+03	5.81e+02
F18	1.50e+04	1.18e+04	2.02e+06	1.81e+06	1.93e+06	1.34e+06	1.45e+06	6.26e+05
F19	6.53e+03	8.53e+03	1.14e+04	1.29e+04	2.57e+04	1.46e+04	4.85e+03	5.07e+03
F20	5.83e+02	1.78e+02	1.50e+03	3.73e+02	1.98e+03	3.90e+02	4.52e+03	6.70e+02
F21	2.66e+02	5.29e+01	4.51e+02	6.43e+01	7.09e+02	7.65e+01	1.48e+03	1.60e+02
F22	1.20e+03	6.26e+02	5.11e+03	9.15e+02	8.18e+03	1.06e+03	1.79e+04	1.41e+03
F23	4.06e+02	1.75e+02	6.16e+02	1.37e+02	9.84e+02	8.16e+01	1.38e+03	1.00e+02
F24	3.33e+02	1.29e+02	9.94e+02	2.06e+02	2.01e+03	2.00e+02	1.98e+03	1.32e+02
F25	4.34e+02	7.90e+01	4.12e+02	2.77e+01	5.53e+02	4.55e+01	7.87e+02	6.42e+01
F26	1.49e+03	6.03e+02	3.75e+03	1.47e+03	6.70e+03	2.09e+03	1.68e+04	2.59e+03
F27	4.63e+02	4.09e+01	5.96e+02	2.96e+01	1.21e+03	2.38e+02	1.37e+03	1.77e+02
F28	5.21e+02	1.44e+02	4.44e+02	3.66e+01	5.07e+02	3.05e+01	5.84e+02	3.05e+01
F29	4.33e+02	1.03e+02	1.38e+03	3.28e+02	2.32e+03	3.64e+02	5.52e+03	6.42e+02
F30	7.32e+05	7.94e+05	1.93e+05	2.46e+05	7.85e+06	4.34e+07	8.49e+05	6.07e+05

**Table 4:** Feature descriptions on different maps for path planning.

Environment	Features	Design Goals
Map 1	Obstacles with large shapes	To evaluate the ability to handle specific passing areas
Map 2	Obstacles with discrete distributions	To evaluate the efficiency of the algorithm
Map 3	Obstacles with concave shapes	To evaluate the ability to handle concave obstacles

**Table 5:** Comparisons with different existing algorithms and statistical tests.

Map	Algorithm	Path Length		Run Times (ms)		Speedup
		**Mean ± Std ↓**	***p*** **-Value ↓**	**Mean ± Std ↓**	***p*** **-Value ↓**	**Mean ↑**
Map 1	A * [45]	49.18 ± 0.00	3.02e-11	1.81 ± 1.30	1.21e-12	321%
	SPSO [37]	57.20 ± 10.17	8.91e-02	2.56 ± 2.34	3.01e-11	495%
	IACO-IABC [38]	58.18 ± 0.00	3.35e-11	1.68 ± 1.41	3.01e-11	291%
	GDRRT * [39]	58.48 ± 5.25	4.98e-04	2.20 ± 1.12	4.07e-11	412%
	GBAS (Ours)	55.67 ± 2.88	-	0.43 ± 0.17	-	-
Map 2	A * [45]	54.60 ± 0.00	1.33e-08	1.29 ± 0.29	4.20e-10	135%
	SPSO [37]	60.41 ± 10.39	1.44e-02	1.60 ± 0.78	3.02e-11	191%
	IACO-IABC [38]	61.60 ± 0.00	2.84e-08	1.98 ± 1.21	3.02e-11	260%
	GDRRT * [39]	61.43 ± 2.95	5.32e-03	1.04 ± 0.46	1.19e-06	89%
	GBAS (Ours)	59.10 ± 3.53	-	0.55 ± 0.16	-	-
Map 3	A * [45]	57.43 ± 0.00	7.47e-10	1.20 ± 0.11	5.09e-06	46%
	SPSO [37]	62.89 ± 8.65	2.17e-01	2.61 ± 1.51	3.02e-11	218%
	IACO-IABC [38]	63.67 ± 0.00	7.87e-11	1.84 ± 1.31	3.02e-11	124%
	GDRRT * [39]	62.49 ± 3.82	5.08e-03	0.99 ± 0.32	2.92e-02	21%
	GBAS (Ours)	61.59 ± 6.41	-	0.82 ± 0.40	-	-

**Table 6:** Comparison of computational time with the benchmark.

Target $v_{\max}$	Method	Mean Times ↓ (ms)	Speedup ↑
1 m/s	Ji et al. [8]	0.96	233%
	Ours	0.18	-
2 m/s	Ji et al. [8]	1.41	555%
	Ours	0.22	-

**Table 7:** Comparison of real-time performance of different models for object detectors.

Model	Backbone	Epochs	FPS ↑	Params ↓ (M)
YOLOv5-L [55]	-	100	42	46
YOLOv6-L [56]	-	100	50	35
YOLOv7-L [57]	-	100	38	36
Ours	-	100	62	43
DINO-Deformable-DETR [58]	R50	36	50	46
RT-DETR-R50 [59]	R50	72	6	42
RT-DETR-L [59]	HGNetv2	72	14	32

**Table 8:** Comparison of key detection performance of different models for object detectors.

Model	GFLOPs ↑	Precision ↑	Recall ↑	F1-Score ↑
YOLOv5-L [55]	109	68.6	87.4	0.77
YOLOv6-L [56]	151	64.5	88.2	0.75
YOLOv7-L [57]	104	70.7	90.4	0.79
Ours	164	85.2	92.7	0.89
DINO-Deformable-DETR [58]	276	69.2	88.6	0.78
RT-DETR-R50 [59]	135	88.2	92.1	0.90
RT-DETR-L [59]	108	87.5	92.4	0.89

## Data Availability

Data are contained within the article. The raw data supporting the conclusions of this article will be made available by the authors on request.
